# Genome-wide characterisation of the Gcn5 histone acetyltransferase in budding yeast during stress adaptation reveals evolutionarily conserved and diverged roles

**DOI:** 10.1186/1471-2164-11-200

**Published:** 2010-03-25

**Authors:** Yongtao Xue-Franzén, Anna Johnsson, David Brodin, Johan Henriksson, Thomas R Bürglin, Anthony PH Wright

**Affiliations:** 1 School of Life Sciences, Södertörn University, Huddinge, SE-141 89, Sweden; 2 Center for Biosciences, Department of Biosciences and Nutrition, Karolinska Institute, Huddinge, SE-141 83, Sweden

## Abstract

**Background:**

Gcn5 is a transcriptional coactivator with histone acetyltransferase activity that is conserved with regard to structure as well as its histone substrates throughout the eukaryotes. Gene regulatory networks within cells are thought to be evolutionarily diverged. The use of evolutionarily divergent yeast species, such as *S. cerevisiae *and *S. pombe*, which can be studied under similar environmental conditions, provides an opportunity to examine the interface between conserved regulatory components and their cellular applications in different organisms.

**Results:**

We show that Gcn5 is important for a common set of stress responses in evolutionarily diverged yeast species and that the activity of the conserved histone acetyltransferase domain is required. We define a group of KCl stress response genes in *S. cerevisiae *that are specifically dependent on Gcn5. Gcn5 is localised to many Gcn5-dependent genes including Gcn5 repressed targets such as *FLO8*. Gcn5 regulates divergent sets of KCl responsive genes in *S. cerevisiae *and *S. pombe*. Genome-wide localization studies showed a tendency for redistribution of Gcn5 during KCl stress adaptation in *S. cerevisiae *from short genes to the transcribed regions of long genes. An analogous redistribution was not observed in *S. pombe*.

**Conclusions:**

Gcn5 is required for the regulation of divergent sets of KCl stress-response genes in *S. cerevisiae *and *S. pombe *even though it is required a common group of stress responses, including the response to KCl. Genes that are physically associated with Gcn5 require its activity for their repression or activation during stress adaptation, providing support for a role of Gcn5 as a corepressor as well as a coactivator. The tendency of Gcn5 to re-localise to the transcribed regions of long genes during KCl stress adaptation suggests that Gcn5 plays a specific role in the expression of long genes under adaptive conditions, perhaps by regulating transcriptional elongation as has been seen for Gcn5 in *S. pombe*. Interestingly an analogous redistribution of Gcn5 is not seen in *S. pombe*. The study thus provides important new insights in relation to why coregulators like Gcn5 are required for the correct expression of some genes but not others.

## Background

Evolutionary changes frequently involve modifications to gene regulatory programs. Several lines of evidence indicate the importance of transcriptional regulation in evolution. First, comparative studies have shown that *cis*-acting regulatory elements that are binding sites for regulatory transcription factors evolve rapidly [1-3]. Second, the proportion of genes encoding transcription factors is higher in genomes encoding larger proteomes consistent with the disproportionately higher regulatory requirements of more complex organisms [4-7], For example, the *nematode Caenorhabditis elegans *has about 100 genes encoding homeobox transcription factors [8] compared to 7 such genes in yeast (*S. cerevisiae*) [4,9], while the total proteome in the nematode is less than 4-fold larger than that in yeast. Third, several yeast species whose common ancestor has undergone whole genome duplication have preferentially maintained paralogous pairs of transcription factors during the re-haploidisation process that ensued, suggesting that transcription factors have a higher than average evolutionary potential [4]. Finally, it has been suggested that non-coding RNA, which is much more abundant in complex organisms with large genomes, plays important roles in regulating the transcription levels of genes [10]. More recently it has been suggested that protein interactions between transcription factors and co-regulator proteins they recruit to target genes may also be important for evolutionary potential [11,12]. Thus, evolutionary changes in the interactions of transcription factors with DNA binding sites and the co-regulators they recruit would predict that the involvement of co-regulator proteins in different regulatory pathways should be evolutionarily diverged.

Gcn5 is a phylogenetically conserved transcriptional co-regulator that is found throughout the eukaryotes. Gcn5 was the first transcriptional co-regulator protein shown to contain a histone acetyltransferase (HAT) activity [13] and it is the catalytic subunit of several related HAT complexes, notably the SAGA complex [14]. These complexes acetylate lysine residues in chromatin, primarily within the N-terminus of histone H3 [15]. The subunit composition of the SAGA complex is highly conserved across the large evolutionary distance between *S. cerevisiae *and *S. pombe *[16]. The SAGA complex is a direct target for recruitment by transcriptional activators *in vitro *[17,18] and *in vivo *[19] and is physically associated to a greater or lesser extent with the promoter and/or transcribed regions of many expressed genes in *S. cerevisiae *[17] and *S. pombe *[20]. The HAT domain is the most highly conserved part of Gcn5 and it has been shown to be inter-changeable between humans and yeast [21]. Gcn5 is thus structurally conserved throughout evolution and appears to function in a conserved fashion by acetylating a conserved set of lysine residues in target proteins. Interestingly, Gcn5 has also been reported to be important for regulation of stress-response genes in budding and fission yeasts [22,23], suggesting that some physiological roles of Gcn5 may also have been conserved but this has not been studied systematically.

Yeasts offer a powerful system for comparative studies because highly divergent organisms can be cultured and manipulated under comparable environmental conditions [24]. Our previous studies show a specific role of fission yeast Gcn5 in programming a subset of stress response genes in *S. pombe *[22]. To further understand functional and evolutionary aspects of Gcn5, we here study the equivalent stress responses in the evolutionarily diverged budding yeasts *S. cerevisiae *and *S. kluyveri*. We show that Gcn5 is required for a common set of stress responses in the budding and fission yeasts. We further report results from a genome-wide study of Gcn5's role in the KCl stress response in *S. cerevisiae *that is comparable to our previous studies in *S. pombe*. The results reveal interesting novel insights with regard to the function of Gcn5 in *S. cerevisiae *and show that it regulates a different set of stress genes compared to those identified in *S. pombe*. Further, we show that Gcn5 is located throughout the transcribed regions of many *S. cerevisiae *genes as has also been shown for *S. pombe *[20], but that there are also mechanistic differences in Gcn5 action between the two species. The study provides an interesting view of the interface between aspects of a transcriptional regulator that are highly conserved and their functionally divergent applications in evolutionarily distant species.

## Results and Discussion

### Gcn5 is required for several common stress responses in divergent yeast species

**Figure 1:**
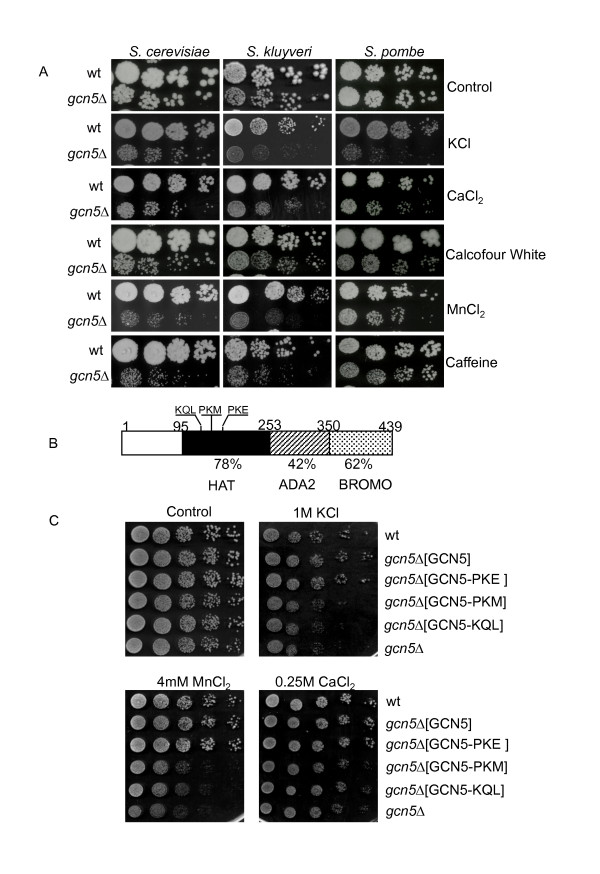
**Gcn5 is required for several common stress responses in divergent yeast species and defects in its conserved histone acetyltransferase domain cause stress sensitivity**. (A) Gcn5 is required for efficient growth under a common set of environmental stress conditions in evolutionarily divergent yeast species. The figure shows serial dilutions of wild type (wt) or mutant (gcn5Δ) yeast cells spotted on normal growth media (control) or media containing stress inducing levels of KCl, CaCl_2_, Calcoflour White, MnCl_2 _and caffeine. (B) The domain structure of Gcn5 showing the highly conserved histone acetyltransferase (HAT). The diagram shows the HAT, the Ada2-interaction domain (ADA2) and the Bromo domain (Bromo) domains defined previously for the *S. cerevisiae *protein [25]. Residue numbers equivalent to domain boundaries are shown. The extent of domain identity between *S. cerevisiae *and *S. pombe *is shown (%). The approximate position of each mutant triple-alanine substitution mutant, starting at residues 126, 129 and 132 respectively, is shown. (C) The HAT activity of Gcn5 is required for Gcn5 dependent stress responses. Cell plating assay performed as in Fig. 1A showing *S. cerevisiae gcn5Δ *cells carrying pRS316 plasmids expressing either wild type *GCN5 *or *GCN5 *with substitution mutations in the HAT domain as indicated in (B). Wild type (wt) and *GCN5*^- ^(gcn5*Δ*) cells were used as controls.

To compare the physiological roles of Gcn5 over a large evolutionary interval, we chose to compare the phenotype of *gcn5Δ *mutants in *S. cerevisiae *and its distant budding yeast relative *S. kluyveri*, using a range of environmental stress conditions, including several conditions where Gcn5 dependence has previously been demonstrated in *S. pombe *[22]. First, we replaced the *GCN5 *gene from wild type *S. kluyveri *with a standard KAN-MX cassette as described in the Materials and Methods. Fig. 1A shows the result of testing the phenotypes of *gcn5Δ *mutants of the three yeast species under different environmental-stress conditions. *gcn5Δ *mutants in *S. pombe *are not distinguishable from wild type cells on rich medium as shown previously [22], but they show a mild growth deficiency in *S. cerevisiae and S. kluyveri*. We observed that Gcn5 was required in *S. cerevisiae *and *S. kluyveri *for adaptation to stresses mediated by KCl and CaCl_2 _as previously observed in *S. pombe *[22]. Gcn5 is also required in all three species for other stress conditions, such as Calcoflour White, MnCl_2 _and Caffeine. These common stress sensitivity phenotypes suggest a specific phenotypically conserved role for Gcn5 because Gcn5 dependence is not universally observed in the three different species for a range of other stress conditions. These conditions include LiCl, MgCl2, NaCl and sorbitol for which none of the yeasts require Gcn5 for stress adaptation. Also for ethanol, elevated temperature and SDS, Gcn5 appears to be differentially important in the different yeast species (see Additional file 1). Quantitative analysis of all measured colony size differences is shown in Additional File 1. The conserved stress conditions thus provide ideal models for studying divergence and evolvability of gene regulation in evolutionarily distant organisms. To facilitate comparison of Gcn5's role in the conserved stress responses of *S. cerevisiae *and *S. pombe*, it was necessary to further characterize the stress response role of Gcn5 in *S. cerevisiae*.

### Defects in the conserved HAT activity of Gcn5 cause stress sensitivity

The HAT domain of Gcn5 is the most conserved domain with a sequence homology of 67% between budding yeast and human [25]. To test whether Gcn5 HAT activity is important for the stress response we tested the ability of plasmids expressing wild type or mutant *gcn5 *alleles, containing triple alanine substitutions, to rescue the salt sensitivity of *gcn5Δ*. The Gcn5-KQL and Gcn5-PKM substitution mutants have previously been shown to abolish HAT activity, while the Gcn5-PKE mutant maintains full catalytic activity [25]. The relative location of the mutated amino acid residues in the Gcn5 HAT domain is indicated in Fig. 1B. Expression of wild type Gcn5 and Gcn5-PKE restores wild type growth levels during KCl and CaCl_2 _stress while strains expressing the Gcn5-PKM and Gcn5-KQL proteins with defective HAT activity display the same level of stress sensitivity as *gcn5Δ *(Fig. 1C). We conclude that the conserved HAT activity of Gcn5 is required for its role in stress responses.

### Identification of KCl stress response genes

**Figure 2:**
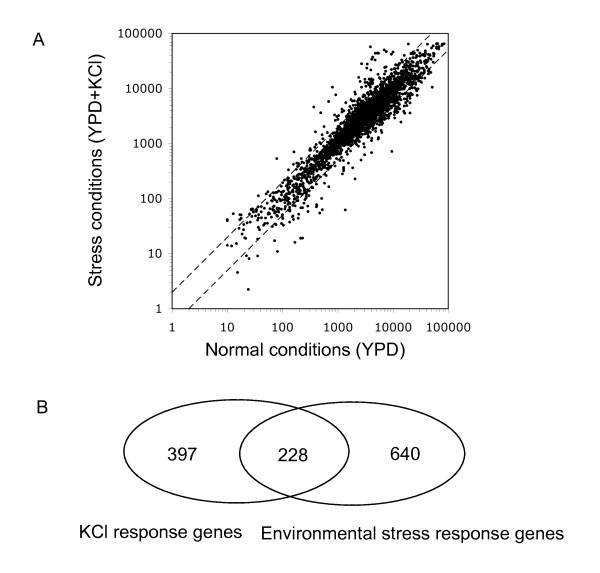
**A subset of KCl response genes are general environmental stress response genes in *S. cerevisiae***. (A) Scatter plot showing the expression levels of genes in the absence (YPD) and presence of KCl (YPD+KCl). The diagonal lines indicate a 2-fold change in expression. (B) A subset of KCl response genes is common environmental stress response genes. Venn diagram of genes differentially regulated during the KCl stress response (left) and genes previously [28] defined as environmental stress response genes (right).

To study how Gcn5 is involved in the KCl stress response of *S. cerevisiae*, we first identified genes with changed expression levels during KCl adaptation in wild type cells. Fig. 2A shows that many genes show either increased or decreased expression in response to KCl (see also gene list in Additional file 2). The gene ontology terms most significantly associated with the sets of KCl-induced and repressed genes are shown in Table 1. The response to KCl-stress conditions is complex but appears to include down-regulation of some general cellular functions (e.g. translation, amino acid biosynthesis) as well as induced expression of genes involved in stress adaptation (e.g. stress response and ion homeostasis). Down regulation of genes involved in protein synthesis during the response of *S. cerevisiae *to other stress conditions has been reported previously [26,27]. More than 30% of genes regulated during KCl adaptation are general environmental stress response genes [28] (Fig. 2B). We found a similar proportion of common stress response genes among the KCl stress response genes of *S. pombe*, as described previously [22]. We also found similar gene ontology categories for KCl-induced genes in both yeast species, such as response to stress/stimuli, oxidoreductase activity and carbohydrate metabolic process.

**Table 1:** Gene Ontology categories enriched in KCl-responsive genes.

GO Category	Total genes†	Changed genes*	P value#
**KCl-induced genes (284)**			

Response to stress	582	42	0.004

Oxidoreductase activity	322	33	2.11e-5

Carbohydrate metabolic process	266	28	5.77e-5

Generation of precursor metabolites and energy	180	18	0.002

Vacuole cell cycle-correlated morphology	165	17	0.002

Cofactor metabolic process	176	17	0.004

Ion homeostasis	121	12	0.013

Glucose metabolic process	88	11	0.003

**KCl-repressed genes (341)**			

Gene expression	1438	150	2.81e-16

Protein metabolic process	1449	120	4.93e-06

Translation	399	101	2.48e-41

Structural constituent of ribosome	221	80	4.804e-45

Ribosome biogenesis and assembly	356	70	5.11e-21

Organic acid metabolic process	386	51	1.28e-08

Carboxylic acid metabolic process	386	51	1.28e-08

RNA binding	476	50	2.04e-05

Oxidoreductase activity	322	32	0.001

Protein-RNA complex assembly	140	29	1.08e-09

Amino acid biosynthetic process	134	23	2.11e-06

Cofactor binding	156	20	0.001

Lyase activity	96	14	0.001

Glutamine family amino acid metabolic process	61	11	0.001

Vitamin binding	61	11	0.001

† Total genes in the GO category.

*Number of KCl induced of reduced genes in the GO category.

*# p*-value from a Fisher's Extract Test (one-sided), statistical considerations are fully discussed in [44].

### Identification of Gcn5 dependent KCl stress response genes

**Figure 3:**
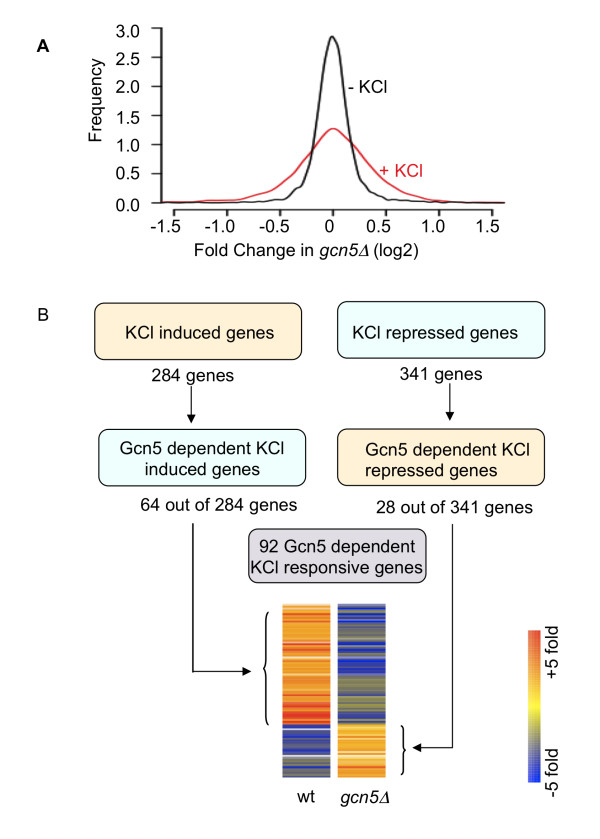
**Identification of Gcn5 dependent KCl stress response genes in *S. cerevisiae***. (A) The Gcn5 dependency of global gene expression increases during KCl induced stress. Graph shows the frequency distribution (y-axis, arbitrary units) of the Gcn5 dependency of genes (x-axis) in the absence (black curve) and presence (red curve) of KCl. The Gcn5 dependency for each gene is expressed as the ratio between the mean transcript level in the gcn5Δ strain in relation to the mean transcript level in the wild type strain. (B) Diagram illustrating how Gcn5 dependent KCl response genes were defined. The selected genes are differentially regulated in wild type cells during KCl stress conditions in a fashion that requires Gcn5 when *gcn5Δ *is compared to wild type during KCl adaptation. Selection criteria are described in the Methods section. The number for of genes in each group is indicated. The heat map shows the fold change values for these genes in the two data sets used to identify them, +KCl/-KCl (wt) and *gcn5Δ*/wild type (*gcn5Δ*).

Next, we investigated the role of Gcn5 in the KCl stress response of *S. cerevisiae*. Comparison of the expression profiles for *gcn5Δ *cells in relation to wild type in the absence and presence of KCl showed that a significant number of genes become more dependent on Gcn5 under KCl stress conditions, as shown by the broader distribution of Gcn5-dependent effects in the presence of KCl (Fig. 3A). Interestingly, there is a fairly equal distribution between genes that require Gcn5 for expression, consistent with its role as a coactivator, and genes that are directly or indirectly repressed by Gcn5. To define a group of Gcn5 dependent KCl responsive genes, we selected a group of 92 genes as summarised in Fig. 3B. Briefly, the group of 92 genes contains KCl-induced genes that show reduced expression in the *gcn5Δ *mutant during KCl stress (64 genes, see Additional file 3) as well as KCl-repressed genes that show increased expression in *gcn5Δ *during KCl stress (28 genes, Additional file 3). The gene ontology categories most significantly associated with Gcn5 dependant KCl response genes (Table 2) include categories that would be expected to play an important role during adaptation to KCl stress conditions, such as ion homeostasis.

**Table 2:** Gene Ontology categories enriched in Gcn5 dependant KCl response genes.

GO Category	**Total genes**†	Changed genes*	P value#
**Gcn5 dependent KCl induced genes (64)**			

Catalytic activity	2271	33	0.016

Oxidoreductase activity	322	13	2.58e-5

Cofactor binding	156	7	0.001

Ion homeostasis	121	6	0.002

Protein folding	97	4	0.019

**Gcn5 dependent KCl-repressed genes (28)**			

Gene expression	1438	14	0.004

Ribonucleoprotein complex biogenesis and assembly	426	9	0.001

RNA processing	448	6	0.019

Translation factor activity nucleic acid binding	58	4	1.64e-4

Iron ion binding	106	4	0.002

Protein-RNA complex assembly	140	4	0.004

Glutamine metabolic process	21	3	1.3e-4

Flocculation	11	2	0.001

† Total genes in the GO category.

*Selection criteria see methods.

*# p*-value from a Fisher's Extract Test (one-sided), statistical considerations are fully discussed in [44].

### Characterization of direct Gcn5 target genes

**Figure 4:**
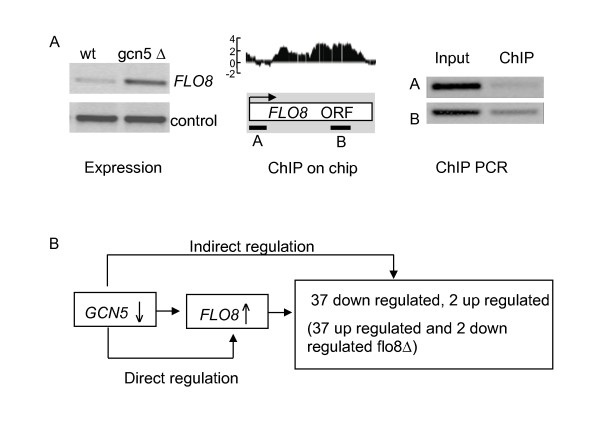
**Gcn5 is associated with the transcribed region of the *FLO8 *gene and causes its repression during KCl stress**. (A) Verification of *FLO8 *regulation and binding by Gcn5. The relative expression level of *FLO8 *gene in wild type and gcn5Δ cells under KCl stress conditions is shown (left panel) in relation to a control gene, YDL212W. Middle panel: tiling array data visualized by IGB showing relative enriched association of Gcn5-myc with *FLO8 *in immunoprecipiated material normalized against input material. The arrow shows the direction of transcription. A and B represent the location of the amplified binding region and control region used for semi quantitative PCR for ChIP, Right panel: semi quantitative PCR validation of Gcn5 association with *FLO8 *showing the level of Gcn5-myc associated chromatin in relation to the input for regions A and B as designated in the middle panel. (B) Diagram showing how deletion of Gcn5 causes indirect regulation of *FLO8 *target genes. *FLO8Δ *vs wild type microarray data are from ([32] GSE4654).

We validated several Gcn5-dependent KCl response genes by semi-quantitative PCR (Additional file 4A). This group includes a membrane transporter *VMR1 *(YHL035C) and a heat-shock-response gene *SSA4 *(YER103W). The latter is consistent with a recent report that Gcn5 and Elp3 induced histone H3 acetylation is important for regulating Hsp70 expression [29]. Interestingly, we also confirmed that the *FLO8 *gene, which encodes a transcriptional regulator, is up-regulated in *gcn5Δ *(Fig. 4A, left panel). This is typical of many Gcn5 dependent genes and its dependence on Gcn5 for its stress-regulated repression is of interest because Gcn5 is generally regarded as a transcriptional activator. One possibility is that up-regulation of *FLO8 *in *gcn5Δ *could be an indirect effect of the Gcn5 defect. To identify direct targets of Gcn5 we performed ChIP-on-chip experiments to identify the genome-wide localisation of myc-tagged Gcn5 using high resolution tiling arrays under the KCl stress conditions in which Gcn5 is required for *FLO8 *expression. This analysis showed reproducible interaction of Gcn5 with the *FLO8 *transcribed region under KCl stress conditions, suggesting direct repression of *FLO8 *expression by Gcn5 (Fig. 4A, middle panel). Direct association of Gcn5 with *FLO8 *was further confirmed by semi-quantitative PCR (Fig. 4A, right panel), The antibody used for precipitation of Gcn5-myc is specific because no signals were detected either in the absence of antibody or in a strain lacking the specific epitope tag in the presence of antibody (see Additional file 4B). We conclude that Gcn5 functions as a negative regulator for *FLO8 *under KCl stress conditions. A repressive role of the Gcn5-containing SAGA complex has been reported previously. In *S. cerevisiae*, Gcn5 is required for repression of *ARG1 *on rich media where expression is not required but the role of Gcn5 switches on minimal media, where it is required for induction of *ARG1 *[30]. A similar function of Gcn5 and the SAGA complex has been reported in *S. pombe*, in which Gcn5 represses *ste11 *and *mei2*, while another SAGA subunit, *Spt8 *is required for their activation under inducing conditions [31].

As expected, we observed enriched association of Gcn5-myc on many Gcn5-dependent genes. However, there are other Gcn5-dependent genes for which we do not observe enrichment of Gcn5. The detection of transcription factor genes, such as *FLO8*, among the direct Gcn5 targets suggests that many of the indirect target genes might be regulated by such transcription factors. This is true for Flo8, where 39 of 44 Flo8 target genes identified previously [32] are dependent on Gcn5 in our study. Of these 39 genes, 37 were up-regulated and 2 were down-regulated in the absence of *FLO8*. In *gcn5Δ *cells, where *FLO8 *is up-regulated, these genes show the opposite regulatory patterns as would be expected if their Gcn5 dependence is an indirect consequence of defects in the direct regulation of *FLO8 *by Gcn5 (Fig. 4B).

### Changes in the pattern of Gcn5 localization under stress conditions

**Figure 5:**
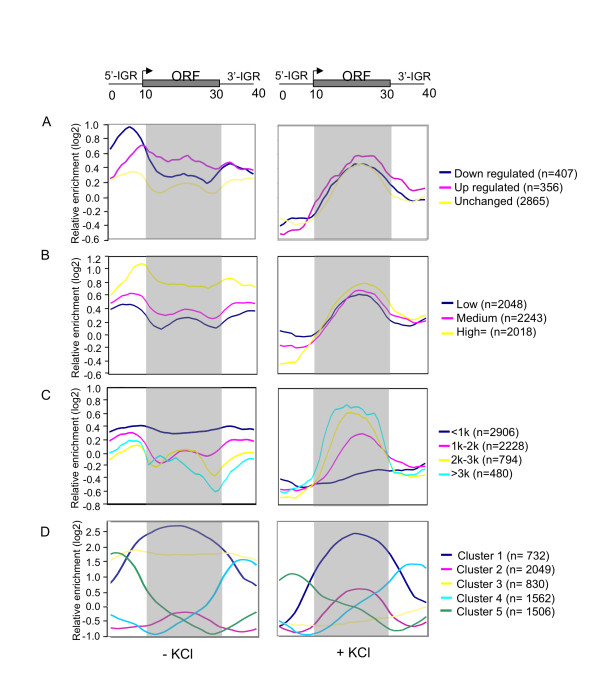
**Genome-wide re-distribution of Gcn5 when cells are exposed to KCl stress**. Average gene analysis shows the average gene distribution of Gcn5-myc enrichment in the absence (left panel) and presence of KCl (right panel). (A) Average Gcn5-myc localisation is shown for groups of up-regulated, down-regulated and unchanged genes. (B) Gcn5-myc is preferentially enriched in highly expressed genes in the absence and presence of KCl stress. Genes are grouped according to their expression level as described in Methods section. (C) Gcn5-myc is preferentially enriched on long genes under KCl stress but is under-represented on such genes in the absence of stress. Genes are grouped according to their ORF length. (D) Gcn5 redistributes between groups of genes with different Gcn5-binding patterns in response to KCl stress. The graphs show the average distribution of Gcn5 in 5 different K-means clusters that were selected to reveal gene groups with different patterns of Gcn5 localisation throughout the genes. The shift of Gcn5 association between groups in response to KCl stress is shown.

To analyse the genome-wide pattern of Gcn5 localisation as well as whether the localisation pattern changes upon KCl stress, we analysed the distribution of Gcn5 within genes in the absence and presence of KCl using average gene analysis. In this method, data for each gene is mapped onto a hypothetical gene of average length, thus allowing studies of average Gcn5 distribution across a model gene for different groups of genes. First we examined the average localisation of Gcn5 in sets of genes that are either up-regulated, down-regulated or unchanged in cells lacking Gcn5 (Fig. 5A). In the absence of KCl, Gcn5 tends to be most abundant at or close to the promoter region (Fig. 5A left panel). This is consistent with previous data showing that Gcn5 is localised predominately to promoter regions [33] as well as the known pattern of histone acetylation within genes [34,35]. In the presence of KCl, the average localisation of Gcn5 is predominantly within the transcribed region of genes (Fig. 5A right panel). The changed average distribution of Gcn5 to transcribed regions during KCl stress conditions suggests a role of Gcn5 in the transcribed regions of genes under these conditions. Such a role has been suggested for Gcn5 on the *GAL1 *gene where it appears to play a transcription-coupled role in nucleosome eviction in the transcribed region [36] during induction of the gene by galactose. Recently we showed that Gcn5 plays an important role in transcriptional elongation in *S. pombe *[20]. It is thus possible that an elongation-related role of Gcn5 may be conserved throughout the eukaryotes.

In *S. pombe*, Gcn5 tends to be preferentially located within the transcribed regions of highly expressed genes in the absence of stress [20]. To test whether this is so in *S. cerevisiae *we used average gene analysis to test the distribution of Gcn5 in sets of genes containing genes expressed at different levels (Fig. 5B). In the absence of stress Gcn5 tends to be preferentially localised to highly expressed genes but it is not preferentially located in the transcribed region. However, during KCl stress the average localisation of Gcn5 is in the transcribed region but its preference for highly expressed genes under these conditions is marginal and may not be significant.

It was reported previously [36] that a very long coding region expressed from the *GAL1 *promoter was more sensitive to defects in Gcn5 than the normal *GAL1 *gene, which has a shorter coding region. In particular, the level of RNA Polymerase II at the 3'-end of the long-gene coding region was sensitive to defects in Gcn5. This suggests that Gcn5 might be particularly important for transcription of long genes in *S. cerevisiae*. To investigate this at the genome-wide level we used average gene analysis to study the distribution of Gcn5 on gene sets containing genes of different length, both in the absence and presence of KCl stress (Fig. 5C). In the absence of stress (Fig. 5C, left panel) the level of Gcn5 at transcribed regions is inversely related to their length. This pattern is completely changed upon KCl stress, where there is a clear positive correlation between the levels of Gcn5 in the transcribed regions and gene length (Fig. 5C, right panel).

**Figure 6:**
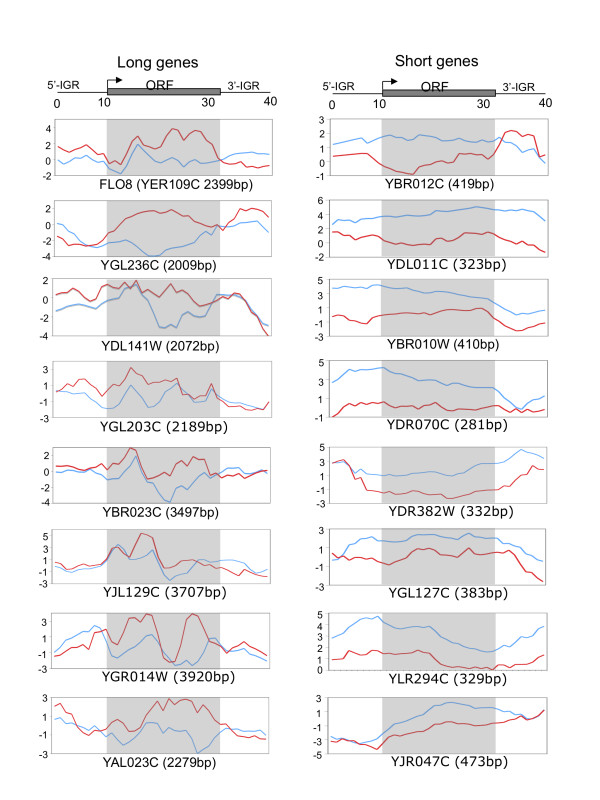
**Altered distribution of Gcn5 on individual long and short genes in response to KCl induced stress**. Examples of Gcn5-myc enrichment in the absence (blue curve) and presence of KCl (red curve) on individual long genes (left panels) and short genes (right panels) are shown. The data plotted for individual genes is taken from the data used for average gene analysis in Fig. 5. Gene length is indicated in parenthesis.

The changes in the average distribution of Gcn5 upon KCl stress could be due to a shift in the location of Gcn5 on individual genes or it could result from enhanced or depleted levels of Gcn5 on specific classes of genes that together make up the observed average localisation patterns. To distinguish between these possibilities, we used K-means clustering to identify the most clearly defined gene sets according to their average Gcn5 localisation pattern (Fig. 5D). Interestingly, we identified five gene clusters for which the average Gcn5 localisation pattern was representative of the majority of individual genes within the cluster (see Additional file 5). Comparison of the average Gcn5 localisation for these clusters in the absence and presence of KCl showed differences that can account for the overall changes in average Gcn5 localisation. Notably, Cluster 2, containing genes for which Gcn5 tends to be located in the transcribed region, shows enhanced Gcn5 association in response to stress. Furthermore, Cluster 3, containing genes with an even distribution of Gcn5 throughout the gene, shows a strong reduction in the average level of Gcn5 association. The changes in these two clusters are the main reasons for the apparent shift of Gcn5 to transcribed regions during stress. The remaining clusters show smaller changes in the average Gcn5 localisation pattern but the subtle changes that do occur also tend to specifically enhance the level of average Gcn5 association with transcribed regions. Strikingly, Cluster 2 is significantly enriched in long genes (>2000 bp, p = 1.31 E-67); it contains 605 genes that are longer than 2000 bp, representing about half of all the genes within this length category. Cluster 3 is significantly enriched in short genes (<500 bp, p = 5.85 E-47) and includes 301 genes shorter than 500 bp. Thus, adaptation to KCl stress is associated with a net transfer of Gcn5 from short genes, where it tends to be evenly distributed throughout genes, to long genes, in which it is predominantly localised within the transcribed region. Fig. 6 shows the re-distribution of Gcn5 on individual genes chosen from Clusters 2 and 3 in response to KCl mediated stress. The Gcn5 redistribution between different genes thus accounts for the changes in average Gcn5 localisation shown in Fig. 5C.

### Comparative analysis of gene regulation by Gcn5 during stress adaptation

**Figure 7:**
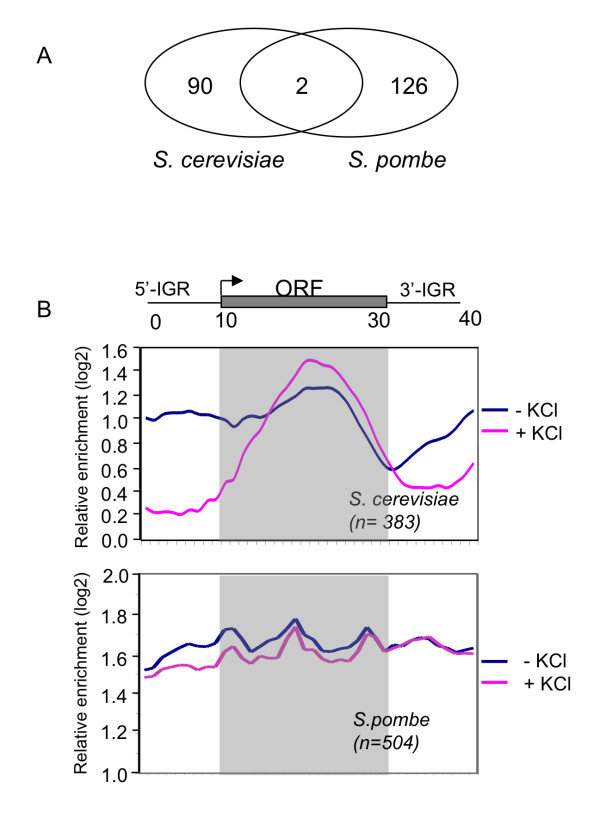
**Gcn5 differs in gene regulation and genome-wide localisation in *S. cerevisiae *and *S. pombe *during KCl stress adaptation**. (A) Gcn5 regulates different sets of KCl responsive genes in *S. cerevisiae *and *S. pombe*. The Venn diagram shows the relationship between gene sets identified in *S. cerevisiae *and *S. pombe*. (B) The altered Gcn5-myc gene distribution pattern between normal and KCl-stress conditions in *S. cerevisiae *is not observed in *S. pombe*. The average gene distribution of Gcn5-myc is shown for genes that are associated with Gcn5 in both the absence and presence of KCl stress. The distribution of Gcn5 is shown for conditions in the absence (blue line) and presence of KCl (red line) for *S. cerevisiae *(average of 383 genes, upper panel) and *S. pombe *(average of 504 genes, lower panel), respectively.

Since gene regulatory networks evolve rapidly (see Introduction), we did not expect Gcn5 to be involved in evolutionarily conserved functional applications even though it is a highly conserved protein at the structural and mechanistic levels. It was therefore of interest that Gcn5 plays a role in adaptation to a common set of stress responses in evolutionarily divergent yeasts (Fig. 1A). To determine whether cross-species conservation is also seen at the level of Gcn5 target genes, we identified a set of Gcn5-dependent KCl response genes in *S. pombe *(123 genes, see Additional file 6) in previously published microarray data [22], using the same criteria as for *S. cerevisiae *data in this work. Gene Ontology analysis showed that several functional categories are enriched in Gcn5 dependent KCl response genes in both yeasts (e.g. transmembrane transporter activity, iron-ion binding, glutamate metabolic process). To compare whether orthologous pairs of genes are involved in Gcn5 dependent adaptation to KCl stress in *S. cerevisiae *and *S. pombe*, we used unambiguous orthologous relationships that have been predicted for 2900 pairs of genes [37], which is about half of all *S. cerevisiae *genes. Comparison of the 92 Gcn5 dependent KCl responsive genes in *S. cerevisiae *with the equivalent group of 123 genes identified in *S. pombe *revealed only 2 cases where both *S. cerevisiae *and *S. pombe *genes were members of an orthologous group, with the same regulation pattern (Fig. 7A). The extent of the overlap is at a level that would be expected by chance and may thus not be statistically significant. The two orthologous pairs are YDL171C-SPAB1E7.07, encoding a glutamate synthase, which has a function in detoxification, and YDR223W-SPAC22H10.11C, encoding a transcriptional corepressor, involved in the regulation of ribosomal protein gene transcription. It was formally possible that the KCl response could be so diverged between the two yeast species at the gene level, that there would be few or no co-regulated orthologous gene pairs that are potential targets for Gcn5. However, this appears not to be the case since there are many orthologous gene pairs that are regulated similarly in response to KCl stress in both *S. cerevisiae *and *S. pombe *(Additional file 7). We conclude that Gcn5 regulates divergent sets of KCl responsive genes in *S. cerevisiae *and *S. pombe*.

Finally, we wanted to find out if there is a similar change in the distribution pattern of Gcn5 when cells are subjected to stress conditions in the two yeasts. To allow comparison with the *S. cerevisiae *ChIP-on-chip data described above we performed analogous ChIP-on-chip experiments to determine the localisation of Gcn5-myc in *S. pombe *during KCl stress adaptation. Equivalent data in the absence of stress have been published recently [20]. We selected groups of genes from *S. cerevisiae *and *S. pombe *that showed a level of Gcn5 enhancement greater than 2.6-fold (log2 1.4) in both the absence and presence of KCl. The localisation pattern in *S. cerevisiae *differs between the two conditions consistent with the results in Fig. 5 (Fig. 7B upper panel), while the pattern for Gcn5-myc in *S. pombe *is similar under both conditions (Fig. 7B lower panel). The different behaviour of *S. cerevisiae *and *S. pombe *suggests that there are differences in the redistribution behaviour of Gcn5 between the two yeasts.

The results published here provide new insights regarding the role of coregulator proteins such as Gcn5 during physiological adaptation in the budding yeast, *S. cerevisiae*. The results also provide insights about how the enzymatically conserved Gcn5 protein is utilized divergently in evolutionarily divergent organisms. Although Gcn5 plays a role in a set of stress responses that seems to be conserved across the large evolutionary distance between budding and fission yeasts, we find that Gcn5 is important for the expression of divergent sets of genes during adaptation to at least one of these stress conditions. This finding is consistent with existing findings that support the highly evolvable nature of gene regulation networks (see Introduction). The divergent requirement for Gcn5 might thus be due to altered recruitment to target genes as a result of changes in the position or composition of transcription factor binding sites or changes in the repertoire of protein interaction partners available to transcription factor proteins. A recent comparative study provides a similar example between much more closely related species, *S. cerevisiae *and *Candida glabrata *[38]. The gene expression responses to oxidative stress are remarkably conserved between these two species, but the underlying regulatory networks differ. Each species appears to have different response motifs and the oxidative stress response transcription factor (Yap1p in *S. cerevisiae *and Cgap1p in *C. glabrata*) show clear differences in the way they "read" the cis-regulatory elements present in target promoters. Other examples of transcription networks, such as the regulation of S-phase related transcription in budding and fission yeasts by the MBF/SBF transcription factors, show a high level of evolutionary conservation between transcription factors and their target genes [39].

Gcn5 is a structurally and mechanistically conserved protein that functions by acetylating other proteins, notably histone H3, in a specific manner via its HAT domain or by specifically binding to acetylated histone H3-K14 residues via its Bromo domain. As mentioned above the difference in the identity of Gcn5-dependent stress genes in *S. cerevisiae *and *S. pombe *is most likely a result of divergence in the genes to which Gcn5 is recruited in different yeasts, since gene regulation networks are known to evolve rapidly. Over the evolutionary distance separating *S. cerevisiae *and *S. pombe *we would not expect to find any conservation of biological function for the Gcn5 protein. It is thus interesting to consider why Gcn5 function is required for a common set of stress responses in the different yeast species studied here. One possibility is that the evolution of Gcn5 function is constrained by some aspect of its function, other than the target genes it regulates. For example, Gcn5, or perhaps other proteins in the SAGA complex, could be regulated by evolutionarily conserved stress-specific signaling pathways that modulate Gcn5 activity. This mechanism would require some stress genes to be Gcn5 dependent but the identity of such genes could be different in different yeasts. Interestingly, there are known examples in which coregulators are direct targets for signaling pathways [40]. It is also possible that loss of Gcn5 recruitment to stress genes during evolution often creates growth defects during stress that tend to be suppressed by acquisition of Gcn5 recruitment by other genes involved in the same stress response, as a result of natural selection.

The results provide new insights into the mechanistic role played by Gcn5 during stress adaptation, which appear to show both similarities and differences between *S. cerevisiae *and *S. pombe*. We provide further support for the direct role of Gcn5 as a repressor of many genes as has been suggested previously for both *S. cerevisiae *and *S. pombe *(discussed above). The re-distribution of Gcn5 between genes during stress adaptation appears to be more dynamic in *S. cerevisiae *than in *S. pombe*, where the average Gcn5 localisation profiles are similar in the absence and presence of stress. The gene cluster (Cluster 2) showing enhanced association of Gcn5 in the transcribed region during stress adaptation contains about one third of all the genes. 145 of these genes are regulated during KCl adaptation and 199 are dependent on Gcn5. These gene sets are significantly enriched in long genes (p < 0.0005). Therefore, the enhanced Gcn5 binding during KCl adaptation can account for some of the observed regulatory changes and Gcn5 may have a role in transcribed gene regions that is specific for long genes. The role of Gcn5 in transcribed regions is associated with the efficiency of transcriptional elongation in *S. pombe*. This may also be the case in *S. cerevisiae *but further studies are required to test this. Gcn5 has been shown to be important for eviction of nucleosomes from the transcribed region of the *GAL1 *gene [36], which would be consistent with an elongation role in *S. cerevisiae*.

## Conclusions

Gcn5 is an evolutionarily conserved protein that is required for a common set of stress responses across a highly divergent range of yeast species. These responses require the activity of the conserved HAT domain but are mediated by divergent sets of Gcn5 dependent response genes in *S. cerevisiae *and *S. pombe*. Gcn5 is localised to *S. cerevisiae *genes that require its activity for their repression as well as for activation, thus providing support for the suggestion that Gcn5 can function as a direct repressor of gene activity in addition to its characterized role as a transcriptional coactivator. Gcn5 localisation tends to shift from short genes to the transcribed regions of long genes during stress adaptation in *S. cerevisiae*. No such change is detectable in *S. pombe*. In the absence of stress Gcn5 is preferentially localised on highly expressed genes in *S. cerevisiae*, as previously reported for *S. pombe*.

## Methods

### Strains, plasmids and growth conditions

The yeast strains used in this study are listed in Table 3. The *GCN5 *deletion mutation in *S. kluyveri*, was made by first inserting PCR products (c. 500 bp) containing sequences up- and down-stream of the Gcn5 coding sequence into pFA6a-KanMX6 at the BamHI and EcoRI sites, respectively. The primers used are listed in Additional file 8. A DNA fragment containing the KanMX cassette flanked by the up- and down-stream flanking regions was then amplified and transformed into the wild type strain of *S. kluyveri*, Y090. Kanamycin resistant colonies in which the Gcn5 coding region was replaced by the KanMX cassette were selected on plates containing G418 (50 mg/ml) and confirmed by PCR.

**Table 3:** Yeast strains used in this study.

Strain name	Yeast species	Genotype	Source
Fy368	*S. pombe*	*h-, leu-32, ura4-D18, ade6-M210*	Ekwall K
Hu799	*S. pombe*	*h-, gcn5::KAN-MX, leu-32, ura4-D18, ade6-M210*	[22]
By4741	*S. cerevisiae*	*MATa, his3-1, leu2-0, met15-0, ura3-0*	Murén E
By7285	*S. cerevisiae*	*By4741, gcn5::KAN-MX*	Murén E
By4742	*S. cerevisiae*	*MATα, his3-1, leu2-0, lys2-0, ura3-0*	Murén E
By17285	*S. cerevisiae*	*By4742, gcn5::KAN-MX*	Murén E
Y090	*S. kluyveri*	*MATα thr*	Piškur J
HuY090 g	*S. kluyveri*	*Y090 gcn5::KAN-MX*	this work
BQS1350	*S. cerevisiae*	*By4742 Gcn5-MYC13-KanMX6*	[17]
Hu2020	*S. pombe*	*h-, gcn5-myc::KanMX6, leu 1-32, ura4-D18, ade6-M210*	[20]

† Total genes in the GO category.

*Selection criteria see methods.

*# p*-value from a Fisher's Extract Test (one-sided), statistical considerations are fully discussed in [44].

*S. cerevisiae *strains expressing Gcn5 derivatives containing substitution mutations in the HAT domain were made by transferring mutant alleles of *GCN5 *from existing plasmids, PKE-pRS306, PKM-pRS306, KQL-pRS306, YG5-pRS414 [25], to pRS316 [41]. The PvuI fragment from the donor plasmid containing *GCN5 *replaced the equivalent fragment from pRS316 that lacked *GCN5*. The new plasmids, PKE-pRS316, PKM-pRS316, KQL-pRS316, YG5-pRS316 (control containing wild type *GCN5*) were transformed into gcn5Δ deletion strain BY7285 and BY17285 and transformants were selected on SD-ura plates.

*S. cerevisiae *and *S. kluyveri *strains were cultivated in YPD medium (1% yeast extract, 2% bacto peptone and 2% glucose) and *S. pombe *strains were cultivated in YEA medium (0.5% yeast extract, 3% glucose, 0.2% cas-amino acids with 100 mg/L of adenine, uracil and leucine, respectively) All the spotting assays were performed by spotting 5-fold serial dilutions of cells on rich medium supplemented with different stress-inducing compounds as shown in Fig. 1A and Fig. 1C. Concentrations of KCl, CaCl_2_, Calcoflour White, MnCl_2_, and caffeine for *S. cerevisiae *and *S. kluyveri *were 1 M, 0.25 M, 20 ug/ml, 4 mM and 6 mM, respectively. The equivalent concentrations for *S. pombe*, were 1 M, 0.1 M, 2 mg/ml, 2 mM and 8 mM respectively. The growth incubation temperature was 25°C for *S. cerevisiae *and *S. kluyveri *and 30°C for *S. pombe*. For other stress conditions see Additional File 1.

### Gene expression profiling

*S. cerevisiae *wild type and *gcn5Δ *strains were compared by expression profiling under exponential growth conditions at 25°C. To determine the effect of KCl stress on gene expression, cells were treated as described in [22]. For each condition, at least two biological replicates were used. An equivalent number of replicates were analyzed using each of the two possible Cy3/Cy5 dye orientations on double spotted microarray slides (Eurogentec SA Seraing, Belgium) and the results were used to calculate the mean fold change value for each gene for each condition tested. RNA extraction, probe labelling and hybridization were performed as previously described [42]. Slides were scanned using a Bio-rad VersArray ChipReader and quantified with Imagene 4.2 software. All the primary data were normalized by Lowess normalization using GeneSpring software. Regulated genes were defined as genes for which the fold changes exceeded a level equivalent to one standard deviation about the overall population mean and for which the change was statistically significant (p < 0.05). The significance of gene expression changes was assessed using Student's t-test to determine the probability that mean fold change values differ from a ratio of one. The null hypothesis tested was that there is no difference. Unchanged genes were defined as genes having a fold change value less than 1.2. All gene expression profiling data analyzed in this study is available at Gene Expression Omnibus (GEO) http://www.ncbi.nlm.nih.gov/projects/geo under accession number: SuperSeriesGSE 16556/Subset series GSE5218.

### ChIP-on-chip microarray

Two biological replicates are used to study enriched binding sites of Gcn5-myc under normal conditions and 1 M KCl treatment conditions in *S. cerevisiae *and *S. pombe*. The ChIP-on chip experiments were carried out as in [20] except the differences in material in culture medium (*S. cerevisiae*: YPD, *S. pombe*: YES) and array used (GeneChip *S. cerevisiae *Tiling 1.0R, Genechip *S. pombe *tiling 1.0FR). The data are available at GEO under accession number: SuperSeriesGSE 16556/Subset series GSE16514.

#### ChIP-on-chip data analysis

Raw data from Affymetrix (CEL format) were analyzed using Model-based Analysis for Tiling-array (MAT) software [43] with a bandwidth of 250 in order to identify regions enriched in Gcn5. Visualization of data was performed using Affymetrix integrated genome Browser (IGB). Average gene analysis was done as in Johnsson *et al *[20]. ChIP-on-chip data of normal conditions and KCl stress conditions were normalized in order to have the same overall standard deviation and mean value. Gene with different expression levels were assigned into three groups by the mean signal intensities of at least three biological replicates of cDNA wild type cells on double spotted cDNA microarray slides (from SuperSeriesGSE 16556/Subset series GSE5218). K-means cluster analysis was performed using GeneSpring software. The number of clusters and iterations was 5 and 100 respectively. Standard correlation was used as the similarity measure.

#### Verification of gene expression and CHIP-on-chip data with semi-quantitative PCR

To verify the gene expression profiling results, cell treatment and RNA extraction were as previously described [22,42]. Reverse transcription of RNA was carried out using reagents from Fermentas (Cat. No. K1611) according to the manufacturers instructions, followed by PCR. To verify the enriched binding of Gcn5-Myc from CHIP-on-chip data, chromatin immunoprecipitation was carried out as described above without the amplification step. Semi quantitative PCR was used to compare immuniprecipitated material for binding and non-binding regions (indicated in the Fig. 4A as fragment A and B) in relation to input (chromatin extract before IP). The primers used are shown in Additional file 8.

### Gene ontology analysis

GoMiner http://discover.nci.nih.gov/gominer/ was used to find gene ontology (GO) terms that are significantly enriched in selected sets of genes with different expression patterns in relation to the frequency of their occurrence in the set of all genes (*p *≤ 0.02). *p*-values were not corrected for multiple hypothesis testing. The statistical analysis performed by the GoMiner algorithm is fully discussed elsewhere [44].

## Authors' contributions

YX-F designed the experiments and performed the phenotypic studies and most micorarray experiments, as well as HAT functional study and PCR confirmation of regulated genes. She analysed the data, interpreted the results and wrote the manuscript. AJ performed part of microarray experiments and contributed to the writing process. DB, JH and TB provided assistence with programming and analysis of tiling array data. AW conceived the idea, supervised the research project and helped with manuscript writing. All authors read and approved the final manuscript.

## Supplementary Material

Additional file 1 **Conditions tested by spotting assays of wild type and *gcn5Δ *mutants in the three yeast species**. A. Additional stress conditions, which are: LiCl, MgCl_2_, NaCl sorbitol, ethanol, elevated temperature and SDS. B. Table showing the mean colony size (± SD), for each strain in each condition, the size of *gcn5Δ *colonies relative to wild type (also plotted in part C), as well as a *p*-value showing the level of significance of size differences between mutant and wild type colonies. Click here for file

Additional file 2 **KCl response genes in *S. cerevisiae***. Gene list for up and down regulated genes under KCl stress response in *S. cerevisiae *with their ratio changes. Click here for file

Additional file 3 **Gcn5 dependent KCl responsive genes in *S. cerevisiae***. Gene list for Gcn5 dependent KCl response genes in *S. cerevisiae *showing their expression pattern and annotation. Click here for file

Additional file 4 **Verification of Gcn5 dependent KCl response genes by semi-quantitative PCR and negative control experiments for ChIP specificity**. Genes validated include: FLO8 (YER109C), VMR1 (YHL035C), HSP70 (YER103W) and control (YDL212W). Click here for file

Additional file 5 **Gcn5 localisation pattern on genes within the 5 K-means clusters**. Gcn5 localisation pattern on genes within the 5 K-means clusters studied in Fig. 5D in the absence and presence of KCl. Click here for file

Additional file 6 **Gcn5 dependent KCl responsive genes in *S. pombe***. Gene list for Gcn5 dependent KCl response genes with their expression pattern and annotation in *S. pombe*. Click here for file

Additional file 7 **Conserved and diverged regulation pattern of KCl response genes between *S. cerevisiae *and *S. pombe***. Hierarchical cluster analysis to compare the expression changes of KCl regulated genes in *S. cerevisiae *with the changes for their orthologs in *S. pombe *during KCl adaptation. Click here for file

Additional file 8 **PCR primers used in this study**. The list of PCR primers used in this study. Click here for file
