# Supplementary material for: Genome-wide characterisation of the Gcn5 histone acetyltransferase in budding yeast during stress adaptation reveals evolutionarily conserved and diverged roles
Source: BMC Genomics. 2010 Mar 25;11:200. doi: 10.1186/1471-2164-11-200 (PMC2861062; doi:10.1186/1471-2164-11-200)
Supplement: Additional file 1 — Conditions tested by spotting assays of wild type and gcn5Δ mutants in the three yeast species. A. Additional stress conditions, which are: LiCl, MgCl2, NaCl sorbitol, ethanol, elevated temperature and SDS. B. Table showing the mean colony size (± SD), for each strain in each condition, the size of gcn5Δ colonies relative to wild type (also plotted in part C), as well as a p-value showing the level of significance of size differences between mutant and wild type colonies. [file 1471-2164-11-200-S1.PDF]

Additional file 1

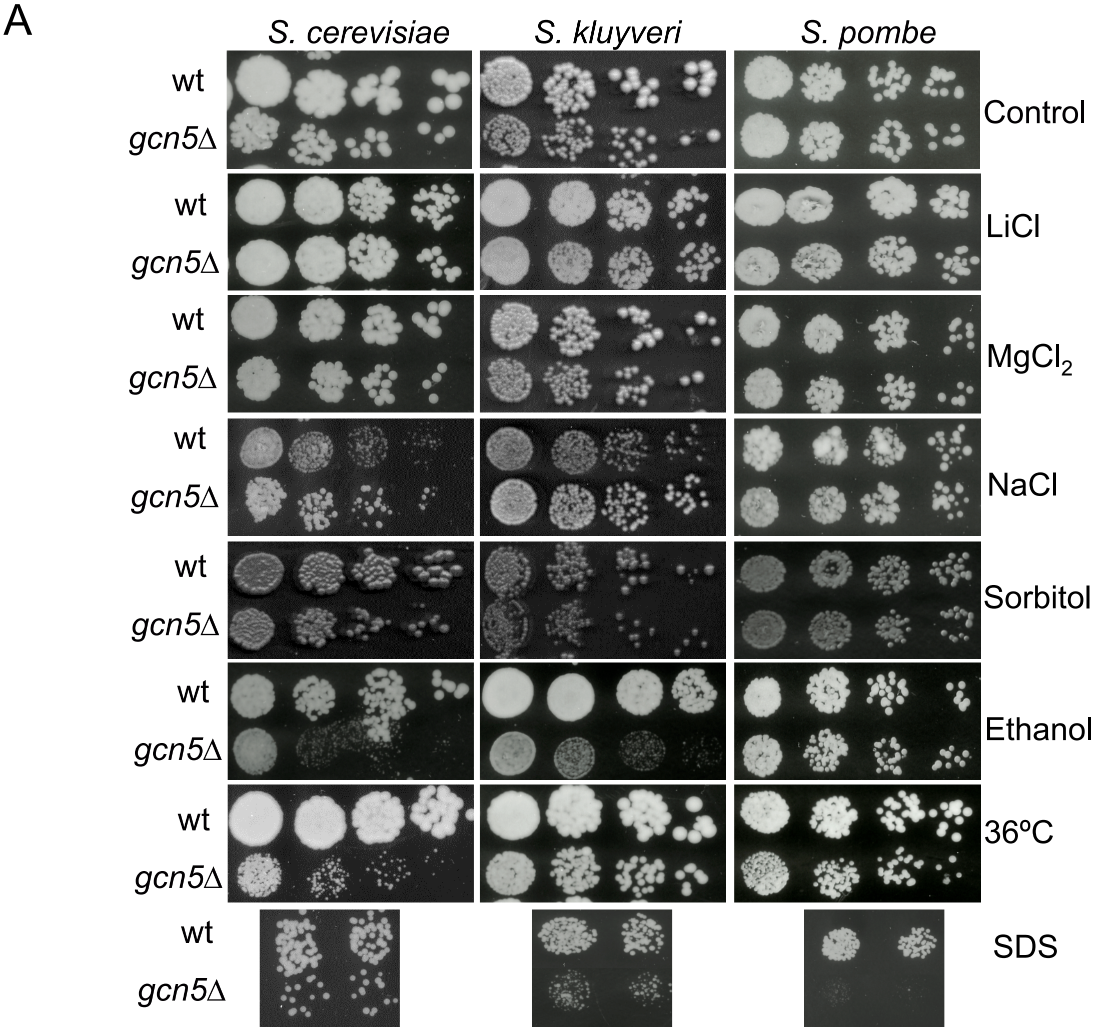

S. cerevisiae and *S. kluyveri* strains were cultivated in YPD m edium (1% yeast extract, 2% bacto peptone and 2% glucose) and *S. pombe* strains were cultivated in YEA medium (0.5% yeast extract, 3% glucose, 0.2% cas-amino acids with 100mg/L of adenine, uracil and leucine, respectively) All the spotting assays were performed by spotting 5- fold serial dilutions of cells on rich medium supplemented with LiCl, MgCl2, NaCl, sorbitol, ethanol, and SDS or incubated at elevated temperature. Concentrations for LiCl, MgCl2, NaCl, sorbitol, ethanol, and SDS for *S. cerevisiae* and *S. kluyveri* were: 10 mM, 0.25M, 0.25M, 1.2M, 6% and 0.02%. The equivalent concentrations for *S. pombe*, were 1 mM, 0.25M, 0.18M, 1.2M and 0.075%. T he growth incubation temperature was 25 ºC for *S. cerevisiae* and *S. kluyveri* and 30 ºC for *S. pombe*. The evaluated temperature was 36 ºC for all three yeast species. Plates were incubated for between 42hr and 120hrs.

Additional file 1

B Extent and significance of colony size differences between gcn5Δ and wild type during different growth conditions.

Conditions	Cell type	*S. cerevisiae* Colony size#	STEDV	Size ratio gcn5Δ/wt	*p* value*	*S. kluyveri* Colony size#	STEDV	Size ratio gcn5Δ/wt	*p* value*	*S. pombe* Colony size#	STEDV	Size ratio gcn5Δ/wt	*p* value*
Rich medium	wt	3.40	0.80	0.69	0.03138857	3.10	0.40	0.64	0.0009144	1.44	0.21	1.04	0.62097053
	gcn5Δ	2.35	0.45			2.00	0.39			1.50	0.18		
KCl	wt	1.89	0.41	0.37	0.00045789	0.58	0.14	0.17	0.00027438	0.59	0.31	0.27	0.02190667
	gcn5Δ	0.71	0.14			0.10	0.02			0.16	0.07		
$CaCl_2$	wt	1.35	0.21	0.29	5.8745E-05	1.78	0.51	0.13	8.2847E-05	1.50	0.11	0.20	4.71E-08
	gcn5Δ	0.39	0.07			0.23	0.10			0.30	0.06		
Calcoflour white	wt	6.45	1.52	0.19	8.8109E-05	1.78	0.43	0.24	1.0121E-05	4.77	1.81	0.15	7.5061E-05
	gcn5Δ	1.25	0.53			0.43	0.18			0.72	0.23		
$MnCl_2$	wt	1.69	0.18	0.08	9.5815E-08	0.49	0.11	0.02	0.00014755	0.96	0.42	0.34	0.00017366
	gcn5Δ	0.14	0.07			<0.01				0.33	0.05		
Caffeine	wt	1.78	0.52	0.13	0.00073573	4.41	0.13	0.04	2.9362E-05	1.35	1.13	0.28	0.00411565
	gcn5Δ	0.25	0.10			0.18	0.20			0.72	0.23		
LiCl	wt	1.39	0.20	1.25	0.14706491	1.37	0.11	0.90	0.22209763	1.52	0.19	0.92	0.18796899
	gcn5Δ	1.73	0.48			1.24	0.23			1.40	0.18		
$MgCl_2$	wt	2.89	0.24	0.58	2.3269E-05	1.82	0.90	1.23	0.52896756	1.32	0.19	1.02	0.84184271
	gcn5Δ	1.69	0.00			2.25	0.00			1.35	0.26		
NaCl	wt	0.27	0.05	1.24	0.37709277	0.55	0.24	2.19	0.00013324	1.28	0.36	0.94	0.48032602
	gcn5Δ	0.34	0.20			1.21	0.48			1.21	0.33		
Sorbitol	wt	2.35	0.32	0.55	0.00353096	0.85	0.71	0.90	0.63452919	0.90	0.20	0.90	0.53490611
	gcn5Δ	1.25	0.19			0.77	0.09			0.81	0.26		
Ethanol	wt	2.25	0.42	0.05	9.2605E-06	1.34	0.29	0.12	2.4795E-07	1.17	0.18	0.82	0.19108823
	gcn5Δ	0.11	0.06			0.16	0.08			0.96	0.25		
36°C	wt	3.48	0.45	0.07	7.349E-08	3.84	1.31	0.59	0.03290923	1.78	0.21	0.65	0.00347013
	gcn5Δ	0.25	0.07			2.25	0.59			1.16	0.26		
SDS	wt	0.98	0.25	0.83	0.2380921	0.75	0.21	0.16	2.4726E-05	0.67	0.18	0.01	5.2661E-05
	gcn5Δ	0.81	0.26			0.12	0.04			<0.01			

\# Mean of colony areas measured in arbitrary units.

* Probability statistic derived from a two-tailed Student's t-test to test the null hypothesis that there is no difference in colony size between gcn5Δ and wild type strains.

C

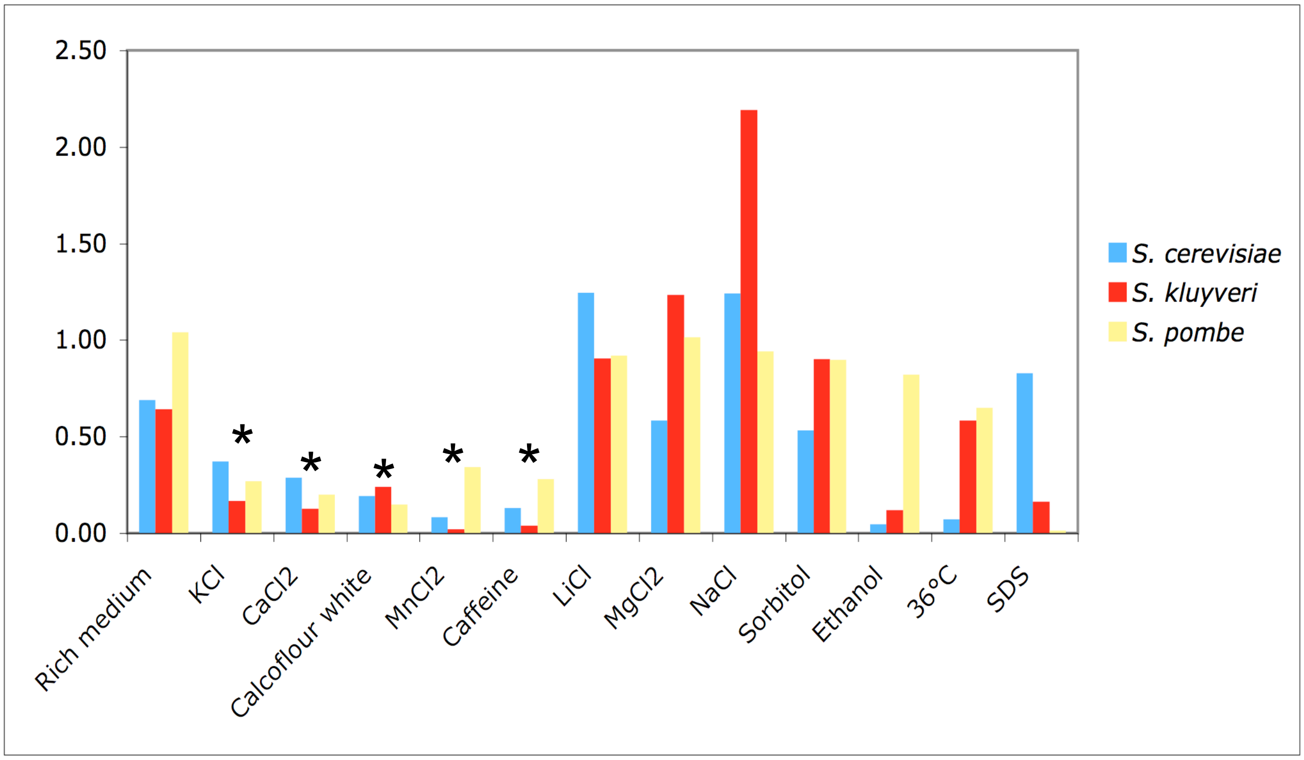

Colony size of gcn5Δ in relation to wild type in different species under different growth conditions. Asterisks show conditions for which gcn5Δ has significantly smaller colonies than wild type for all three species ($p < 0.05$).
