# Supplementary material for: Genome-wide characterisation of the Gcn5 histone acetyltransferase in budding yeast during stress adaptation reveals evolutionarily conserved and diverged roles
Source: BMC Genomics. 2010 Mar 25;11:200. doi: 10.1186/1471-2164-11-200 (PMC2861062; doi:10.1186/1471-2164-11-200)
Supplement: Additional file 4 — Verification of Gcn5 dependent KCl response genes by semi-quantitative PCR and negative control experiments for ChIP specificity. Genes validated include: FLO8 (YER109C), VMR1 (YHL035C), HSP70 (YER103W) and control (YDL212W). [file 1471-2164-11-200-S4.PDF]

Additional file 4

A

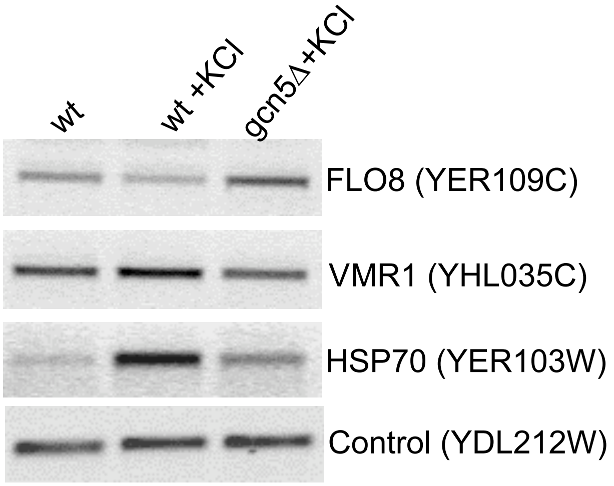

Verification of Gcn5 dependent KCl response genes by semi-quantitative PCR. The names of the verified genes and the conditions of growth are shown.

B

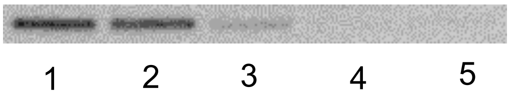

Negative control experiments for ChIP specificity: 1 Input (non-tag control). 2 Input (Gcn5-myc tag). 3 ChIP: Gcn5-myc + Antiboy. 4 ChIP: non-tag control + antibody. 5 ChIP: Gcn5-myc - antibody.
