# Supplementary material for: Genome-wide characterisation of the Gcn5 histone acetyltransferase in budding yeast during stress adaptation reveals evolutionarily conserved and diverged roles
Source: BMC Genomics. 2010 Mar 25;11:200. doi: 10.1186/1471-2164-11-200 (PMC2861062; doi:10.1186/1471-2164-11-200)
Supplement: Additional file 5 — Gcn5 localisation pattern on genes within the 5 K-means clusters. Gcn5 localisation pattern on genes within the 5 K-means clusters studied in Fig. 5D in the absence and presence of KCl. [file 1471-2164-11-200-S5.PDF]

Additional file 5

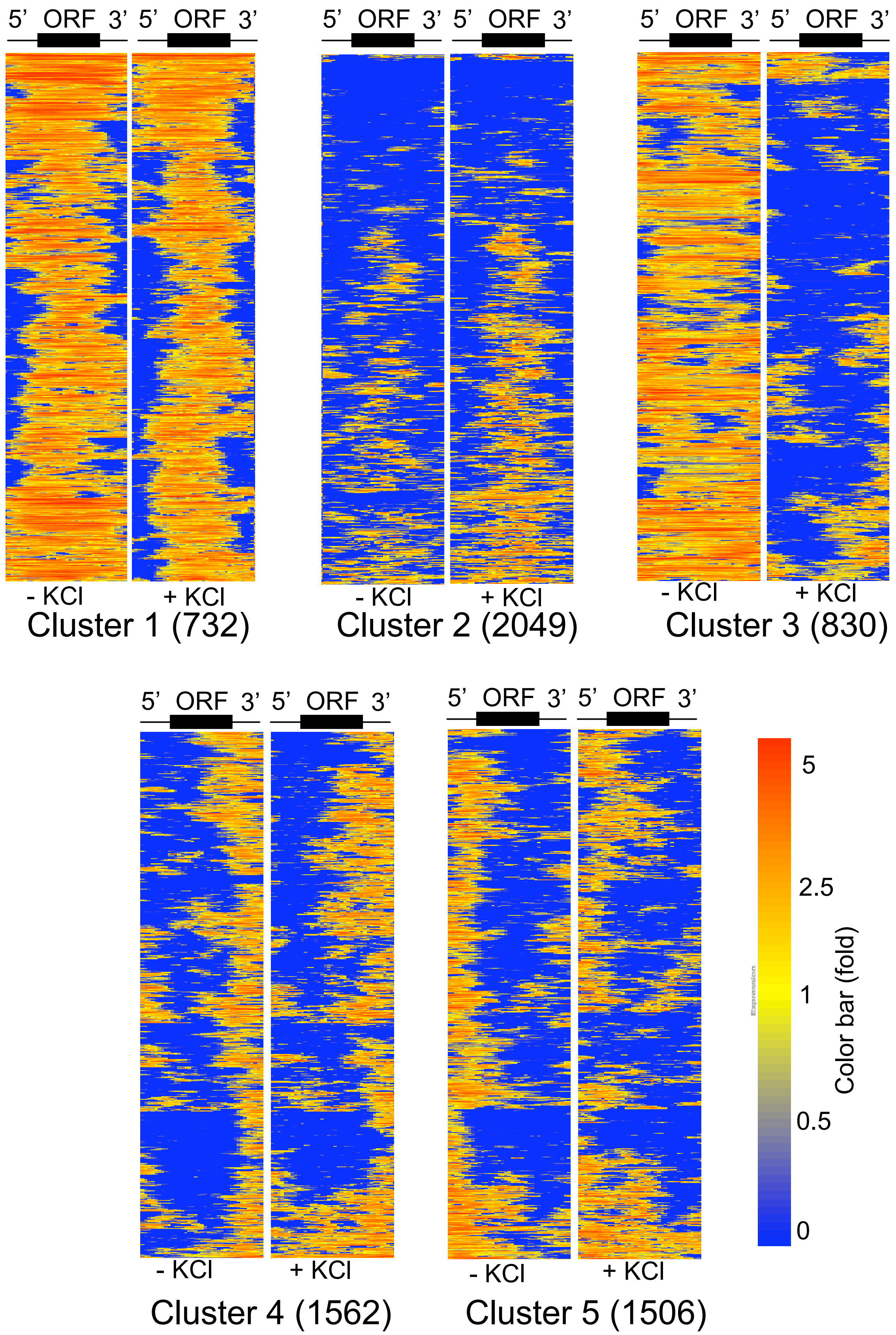

Gcn5 localisation pattern on genes within the 5 K-means clusters studied in Fig. 5D in the absence and presence of KCl is shown by GeneSpring software. Number of genes in each group is indicated. Color bar shows the range of values measured for Gcn5 enrichment.
