# Supplementary material for: Genome-wide characterisation of the Gcn5 histone acetyltransferase in budding yeast during stress adaptation reveals evolutionarily conserved and diverged roles
Source: BMC Genomics. 2010 Mar 25;11:200. doi: 10.1186/1471-2164-11-200 (PMC2861062; doi:10.1186/1471-2164-11-200)
Supplement: Additional file 7 — Conserved and diverged regulation pattern of KCl response genes between S. cerevisiae and S. pombe. Hierarchical cluster analysis to compare the expression changes of KCl regulated genes in S. cerevisiae with the changes for their orthologs in S. pombe during KCl adaptation. [file 1471-2164-11-200-S7.PDF]

Additional file 5

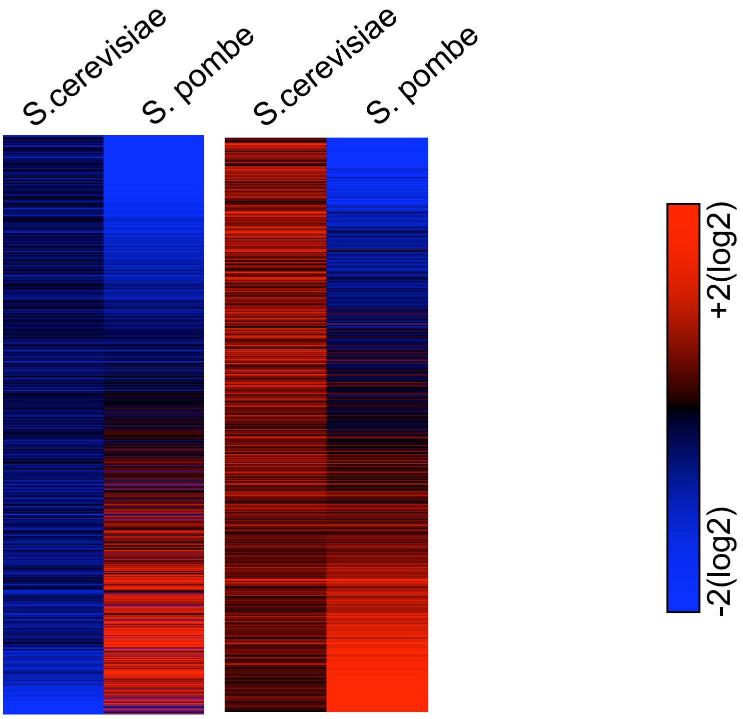

KCl regulated genes show both similarly and differentially regulated in *S. cerevisiae* and *S. pombe*. Hierarchical cluster analysis to compare the expression changes of KCl regulated genes in *S. cerevisiae* with the changes for their orthologs in *S. pombe* during KCl adaptation (similar colour indicates a similar expression pattern). The scale bar indicates the extent of the changes.
